# Real-life comparison of efficacy and safety profiles of two prolonged-release tacrolimus formulations in de novo kidney transplant recipients: 24 months of follow-up

**DOI:** 10.1371/journal.pone.0278894

**Published:** 2023-01-20

**Authors:** Paulina Czarnecka, Kinga Czarnecka, Teresa Baczkowska, Beata Lagiewska, Magdalena Durlik

**Affiliations:** 1 Department of Transplantation Medicine, Nephrology and Internal Diseases, Medical University of Warsaw, Warsaw, Poland; 2 Department of General Surgery and Transplantations, Medical University of Warsaw, Warsaw, Poland; Weill Cornell Medicine, UNITED STATES

## Abstract

**Introduction:**

Calcineurin inhibitors constitute a cornerstone of immunosuppressive therapy in kidney transplant recipients. There are two main formulations of tacrolimus (Tac) which exhibit a prolonged-release mode of action: Advagraf^®^ (MR-4) and Envarsus^®^ (LCPT). However, they are not bioequivalent. Data comparing both once-daily prolonged-release formulations of Tac are insufficient.

**Objective:**

The aim of the study was to compare safety and efficacy profiles of once-daily LCPT and MR-4 formulations of tacrolimus in adult kidney transplant recipients.

**Patients and methods:**

An observational, cohort single-center study was performed. One hundred fifteen kidney transplant recipients transplanted between 2016 and 2019 were enrolled to the study (59 vs 56, Envarsus^®^ vs Advagraf^®^, respectively). Safety and efficacy profiles were assessed.

**Results:**

Patient and graft survival at 12 and 24 months did not differ between the groups. There were no significant differences in serum creatinine at any timepoint. C/D ratio in the LCPT group was significantly higher at 12 and 24 months. Sepsis occurrence was more frequent in MR-4 group at 12 months.

**Conclusion:**

Both prolonged-release formulations of tacrolimus are safe and effective in immunosuppressive therapy in kidney transplant recipients.

## Introduction

Calcineurin inhibitors (CNIs) constitute the mainstay of immunosuppressive therapy for kidney transplant recipients (KTRs). Today, tacrolimus (Tac) is the number one agent of the CNI group that is effective in the prophylaxis of acute kidney allograft rejection. It inhibits T-lymphocyte activation and proliferation, as well as the T-helper-cell-dependent B cell response [1]. Tac is administered to >90% of *de novo* KTRs.

Three main formulations of Tac are readily available in the market. Two exhibit a prolonged-release mode of action: extended-release capsules (MR-4, Advagraf^®^, Astellas) and MeltDose tablets (LCPT, Envarsus^®^, Chiesi). Both share the benefit of patient compliance, which improves graft survival. However, prolonged-release formulations are not bioequivalent and exhibit considerable differences in pharmacokinetic properties and dosage requirements to achieve comparable blood concentration. The MeltDose technology of LCPT is characterized by improved solubility, dispersion in a polymeric matrix, and increased bioavailability. MR-4 is covered with a polymer of ethylcellulose, which slows drug release.

Tac has a narrow therapeutic index; hence, underdosing and overdosing may have serious clinical implications (adverse events [AEs] or episodes of acute organ rejection that affect graft function) [2]. Therefore, close monitoring of Tac blood level and dose adjustment are mandatory for adequate immunosuppressive treatment. The blood concentration levels of Tac and exposure to the drug are influenced by multiple factors, such as age, hemoglobin concentration, gastrointestinal motility, and gene polymorphism. Furthermore, there is evidence to suggest that the concentration/dose (C/D) ratio may be a more accurate indicator of patients’ exposure to Tac compared with trough blood levels and may support early identification of patients at high risk of CNI-induced nephrotoxicity.

Many analyses have shown comparable efficacy and safety profiles of twice-daily immediate-release (IR-Tac) and once-daily prolonged-release formulations. Data comparing once-daily prolonged-release formulations of Tac are insufficient. Moreover, large randomized multicenter studies excluded elderly, pre-sensitized, and retransplanted patients. Observational studies with much less restrictive inclusion criteria involving true KTRs are needed.

## Aim

This was an observational, cohort, single-center study. The objective of this study was to compare the safety and efficacy profiles of once-daily LCPT and MR-4 Tac formulations in adult KTRs followed up for 24 months.

The primary study endpoints were patient and graft survival, renal function evaluated using serum creatinine concentration (Scr) and estimated glomerular filtration rate (eGFR), biopsy-proven acute rejection (BPAR), T-cell mediated rejection (TCMR), antibody-mediated rejection episodes (ABMR), and other histological lesions.

The secondary outcomes were treatment-related AEs, *de novo* donor-specific antibody (DSA) (dnDSA) development, delayed graft function (DGF), primary nonfunction (PNF), new-onset diabetes after transplantation (NODAT), frequency of CNI withdrawal, Tac dose, Tac whole blood concentration (C_0_), and C/D ratio.

### Patients and methods

One hundred forty five KTRs transplanted between 2016 and 2019 were identified. Inclusion criteria were as follows: adult recipients of deceased donor kidneys receiving Tac MR-4 or LCPT. Second or third kidney transplant patients, pre-sensitized and receiving kidney transplants from expanded criteria donors, were included. Patients receiving a kidney from a living donor and simultaneous transplants of the kidney and other organs were excluded from the study.

Ultimately, 115 patients (all Caucasian) were included in this study. All patients received triple immunosuppressive therapy, including once-daily Tac formulation (LCPT or MR-4, 51% n = 59 vs. 49% n = 56, respectively) in combination with prednisone and mycophenolate mofetil. Per the internal institution policy, KTRs alternately receive Advagraf and Envarsus every two months. Once-daily Tac was started directly before transplantation (at half a dose: 0.09 mg/kg of LCPT or 0.1 mg/kg of MR-4) and subsequently at 0.17 mg/kg/day of LCPT or 0.2 mg/kg/day of MR-4 from the next morning. The regimen was followed until day 3, and daily doses were adjusted according to the blood concentration levels of the drug to maintain trough levels of 9–15 ng/mL in the initial post-transplant period and 4–10 ng/mL in the post-transplant maintenance period. The initial transplantation period was defined as 0–30 days post-transplantation. The maintenance transplant period was 30 days onwards. Twenty-eight patients required induction therapy. In our country, there is no protocol for interleukin (IL)-2R blocker induction therapy for patients at low immunological risk. IL-2R blockers are used for patients with moderate immunological risk, whereas thymoglobulin is used for patients with high immunological risk. The details of the induction agent used are summarized in Table 1.

**Table 1:** The baseline characteristics is presented.

		LCPT	MR-4	
Sex				*p* = .838*
M	N (%)	39 (66,1)	36 (64,3)	
F	N (%)	20 (33,9)	20 (35,7)	
Preemptive	N (%)	5 (8,5)	4 (7,1)	*p* = 1**
Hypertension	N (%)	57 (96,6)	50 (89,3)	*p* = .156**
Diabetes	N (%)	15 (25,4)	12 (21,4)	*p* = .643*
KTX				
1	N (%)	51 (86,4)	41 (73,2)	*p* = .076*
2	N (%)	8 (13,6)	8 (14,3)	*p* = .816*
3	N (%)	0 (0,0)	4 (7,1)	*p* = .047**
CMV IgM D	N (%)	0 (0,0)	3 (5,4)	*p* = .112**
CMV IgG D	N (%)	51 (86,4)	48 (85,7)	*p* = 1*
CMV IgM R	N (%)	0 (0,0)	3 (5,4)	*p* = .112**
CMV IgG R	N (%)	48 (81,4)	47 (83,9)	*p* = .808*
DSA A+B	N (%)	4 (6,8)	5 (8,9)	*p* = .739**
DSA DR	N (%)	6 (10,3)	3 (5,4)	*p* = .490**
Induction	N (%)	11 (18,6)	15 (26,8)	*p* = .297*
IL-2R blocker	N (%)	7 (63,6)	6 (40,0)	*p* = .234*
ATG	N (%)	4 (36,4)	9 (60,0)	
Recipient age, *years*	mean (SD)	46.92 (14.46)	50.20 (12.14)	*p* = .189^1^
Donor Age, *years*	mean (SD)	47.63 (15.52)	50.68 (14.82)	*p* = .284^1^
Time on dialysis, months	mean (SD)	22.56 (21.25)	38.64 (36.26)	*p* = .005^1^
WIT, *min*	mean (SD)	40.98 (13.00)	35.39 (19.37)	*p* = .098^1^
CIT, *min*	mean (SD)	1085.19 (432.65)	1096.30 (667.51)	*p* = .941^1^
HLA A mismatches	mean (SD)	1.05 (0.63)	0.98 (0.65)	*p* = .564^1^
HLA B mismatches	mean (SD)	1.17 (0.65)	1.00 (0.63)	*p* = .155^1^
HLA DR mismatches	mean (SD)	0.74 (0.61)	0.71 (0.53)	*p* = .801^1^
PRA	mean (SD)	3.34 (10.68)	3.77 (8.73)	*p* = .815^1^
PRA max	mean (SD)	6.49 (15.95)	7.57 (16.83)	*p* = .727^1^
BMI, *kg/m*^*2*^	mean (SD)	25.06 (3.84)	25.71 (3.93)	*p* = .372^1^
ESDR				*p* = .695^1^
Glomerulonephritis	N (%)	25 (42.4)	22 (39.3)	
ADPKD	N (%)	13 (22.0)	9 (16.1)	
Unknown etiology	N (%)	5 (8.5)	5 (8.9)	
Hypertension	N (%)	4 (6.8)	7 (12.5)	
Diabetes	N (%)	6 (10.2)	3 (5.4)	
Vasculitis	N (%)	1 (1.7)	2 (3.6)	
Congenital urinary tract defect	N (%)	2 (3.4)	2 (3.6)	
Obstructive nephropathy	N (%)	1 (1.7)	2 (3.6)	
Nephrolithiasis	N (%)	1 (1.7)	2 (3.6)	
Heroine nephropathy	N (%)	1 (1.7)	0 (0.0)	
Chronic interstitial nephritis	N (%)	0 (0.0)	1 (1.8)	
HUS	N (%)	0 (0.0)	1 (1.8)	

M, male; F, female; Ktx, kidney transplantation; CMV, cytomegalovirus; D, donor; R, recipient; DSA, donor-specific antibodies; ATG, anti-thymocyte globulin; IL-2R blocker, interleukine-2 receptor blocker; BMI, body mass index; WIT, warm ischaemic time; CIT, cold ischaemic time; HLA, human leucocyte antigen; PRA, panel-reactive antibodies; ESDR, end stage renal disease; ADPKD, autosomal dominant polycystic kidney disease; HUS, haemalytic-uraemic syndrome; SD, standard deviation, min, minute.

*chi^2 test; **Fisher exact test; ^1^ Student’s *t*-test.

Biopsies were performed routinely at baseline and at 3, 12, and 24 months following transplantation, according to the internal protocol (surveillance biopsies) and when clinical rejection occurred (for cause biopsies). Histological specimens containing at least seven glomeruli and one artery were considered diagnostic. Diagnostic biopsies were performed for 83 patients on implantation, 85 patients at 3 and 12 months, and 50 patients at 24 months after kidney transplantation. Changes in histological lesions at 3, 12, and 24 months were calculated in accordance with the semi-quantitative Banff criteria (0–3) by an experienced pathologist [3]. The Banff Chronicity Score (BChS) was calculated. The BChS is based on four parameters: transplant glomerulopathy (‘cg”), tubular atrophy (‘ct’), cortical fibrosis (‘ci’), arterial intimal thickening (‘cv’) with a maximum total score of 12. Surveillance biopsies constituted 72.5% of all procedures performed.

Renal function was evaluated using Scr and eGFR according to the Modification of Diet in Renal Disease study group 4 equation.

Tac trough concentration was assessed in whole blood by immunoassay (C_0_, 24 h after the last Tac dose was administered, prior to the morning dose).

DGF was defined as the need for hemodialysis by day seven after transplantation. NODAT was diagnosed in patients with two fasting plasma glucose measurements of ≥7.0 mmol/L, 2-h plasma glucose levels of ≥11.1 mmol/L during the oral glucose tolerance test or random plasma glucose level of ≥11.1 mmol/L, and symptoms of hyperglycemia.

Clinical outcomes and covariates were evaluated on day 14 and at 1, 3, 6, 12, and 24 months of observation, unless stated otherwise.

Data included demographics, anthropometrics, donor data, peak panel reactive antibodies prior to transplantation, the number of human leukocyte antigen (HLA) mismatches, *de novo* and prior to kidney transplantation DSAs, immunosuppressive therapy, pathological biopsy findings, and patient medical history. Tac doses, whole blood trough levels, vital signs, physical examination, renal function, and biochemical parameters were collected.

### Statistical methods

The symmetry of the distribution was measured by its skewness [4]. Continuous variables were assessed using Student’s *t*-test for independent samples or the Mann–Whitney *U* test when appropriate. The chi-square or Fisher’s exact test was used for categorical and interval data. Whenever chi-square test was used, chi^2 with respective df value was added in addition to p value. In the case only p value is provided, the Fisher’s exact test was used. The chi-square test, Fisher’s exact test, and Spearman’s correlation coefficient were used to study the correlation of the variables. Statistical significance was set at *p* <0.05. Data are reported as mean ± standard deviation (*SD*) unless stated otherwise. The data were analyzed using IBM SPSS Statistics version 25. Institutional Review Board approval was obtained, and the study adhered to the Declaration of Helsinki. The Ethics Committee waived the need of consent from patients as the study was conducted in the retrospective manner.

## Results

### Patient and graft survival

Patient survival in the LCPT and MR-4 groups at 12 months (100% vs. 97.9%, *p* = 1) and 24 months (97.9% vs. 97.9%, *p* = 1) were similar. In the MR-4 group, one death resulted from a systemic fungal infection within 1-year post-transplantation. One patient in the LCPT cohort died of cardiac arrest more than 12 months after kidney transplantation. Graft survival at 12 and 24 months did not differ significantly between the groups and reached 96% vs. 90.2%, *p* = 0.436 and 93.3% vs. 87.5% *p* = 0.488, respectively). One graft loss in the MR-4 population resulted from kidney rupture, and the other from a rejection episode. In the LCPT group, single graft loss was caused by chronic graft glomerulopathy.

### Kidney function

The primary outcome of the study was allograft function, assessed using Scr and eGFR. Similar results were observed at any time point within 24 months of follow-up, regardless of the Tac formulation. The detailed results are presented in Table 2.

**Table 2:** Kidney function within 24 months follow-up post-transplant.

**Creatinine in LCPT and MR-4 groups**							
	LCPT		MR-4				
	*M*	*SD*	*M*	*SD*	*U*	*Z*	*p* ^*1*^
14d	2.57	1.77	2.30	1.53	1409.5	-1.22	.224
1m	2.05	0.99	2.06	1.37	1417.0	-1.17	.241
3m	1.90	1.06	2.05	1.67	1291.5	-1.02	.306
6m	1.64	0.56	1.84	1.34	1126.0	-0.85	.394
12m	1.55	0.49	1.51	0.69	922.5	-1.37	.170
24m	1.55	0.57	1.50	0.57	846.5	-0.50	.619
**eGFR in LCPT and MR-4 groups**							
	LCPT		MR-4				
	*M*	*SD*	*M*	*SD*	*U*	*Z*	*p* ^*1*^
14d	37.80	21.34	40.77	20.42	1450.0	-0.99	.324
1m	41.52	18.80	45.52	22.77	1437.0	-1.06	.289
3m	45.26	17.15	47.98	23.37	1358.0	-0.61	.539
6m	49.49	17.39	51.33	21.93	1149.0	-0.69	.488
12m	51.29	16.05	55.60	19.44	953.0	-1.14	.253
24m	54.18	20.46	54.90	17.69	853.0	-0.44	.660

1the Mann-Whitney U test.

DGF occurred in 11.9% and 14.3% of the patients in the LCPT and MR-4 groups (*p* = 0.785). The mean DGF duration was 13 (5.5) vs. 51 (72.4) days, respectively t (13) = -1,389, *p* = 0.189. The great disparity in DGF between cohorts primarily stems from an exceptionally prolonged DGF of 236 days for the subject from the MR-4 group, with hemolytic uremic syndrome as the primary cause of end-stage renal disease and PNF of his second kidney transplant. PNF rates were comparable, although infrequent, 3.4% n = 2 vs 5.4% n = 3, *p* = 0.674.

### BPAR and histopathological profile

Within 2 years of follow-up, 19 and 16 BPAR episodes were diagnosed (LCPT and MR-4 groups, respectively, chi^2(1)*p* = 0.539). In three patients vs. two patients (LCPT and MR-4 groups, respectively,) more than one episode of BPAR was reported. TCMR rates were similar (26.9% vs. 20.8% at 24 months, chi^2(1), *p* = 0.499, respectively); steroid-resistant TCMR requiring anti-thymocyte globulin (ATG) administration occurred in two patients from the MR-4 group at 3 months. Most TCMR episodes were acute interstitial, according to the Banff criteria.

ABMR at 24 months was identified in 11.5% vs. 11.3% of the patients (chi^2 (1), *p* = 1).

DSAs were tested when BPAR was suspected. dnDSA levels at 24 months were comparable: 28.6% vs. 23.3%, chi^2(1) = 0.217, *p* = 0.649, respectively in the LCPT and MR-4 groups).

Arterial hyalinosis (ah) and tubular atrophy (ct) were more prominent in the biopsy specimens at 3 months in the MR-4 group (*p* = 0.061, *p* = 0.060, respectively). The remaining histopathological findings were specified elsewhere and did not differ between the groups (Table 3).

**Table 3:** Histological data at baseline, 3, 12 and 24 months after transplantation. Biopsy reports were available for 83 patients on implantation (41 –LCMPT, 42 –MR-4), 85 patients at 3 and 12 months (46 –LCPT, 39 –MR-4 and 42 –LCPT, 42 –MR-4, respectively), and 50 patients at 24 months after kidney transplantation (26-LCPT, 24 –MR-4).

			LCPT	MR-4	the Mann Whitney *U* test		
ah_0	0	*N(%)*	28 (68.3)	24 (57.1)	*U* = 742,5 *p* = .210		
		
	1	*N(%)*	9 (22.0)	9 (21.4)			
		
	2	*N(%)*	4 (9.8)	9 (21.4)			
		
ah_3	0	*N(%)*	30 (65.2)	17 (43.6)	*U* = 706 *p* = .061					
		
	1	*N(%)*	7 (15.2)	10 (25.6)						
		
	2	*N(%)*	9 (19.6)	12 (30.8)						
		
ah_12	0	*N(%)*	18 (41.9)	19 (45.2)	*U* = 898.5 *p* = .966		
		
	1	*N(%)*	11 (25.6)	8 (19.0)			
		
	2	*N(%)*	13 (30.2)	14 (33.3)			
		
	3	*N(%)*	1 (2.3)	1 (2.4)			
		
ah_24	0	*N(%)*	6 (23.1)	5 (20.8)	*U* = 260 *p* = .274		
		
	1	*N(%)*	10 (38.5)	5 (20.8)			
		
	2	*N(%)*	10 (38.5)	14 (58.3)			
		
			LCPT	MR-4	the Mann Whitney *U* test
cg_0	0	*N(%)*	38 (92.7)	41 (97.6)	*U* = 818,5 *p* = .297		
		
	1	*N(%)*	3 (7.3)	1 (2.4)			
		
cg_3	0	*N(%)*	40 (87.0)	31 (79.5)	*U* = 830 *p* = .358		
		
	1	*N(%)*	6 (13.0)	8 (20.5)			
		
cg_12	0	*N(%)*	25 (58.1)	23 (54.8)	*U* = 864 *p* = .693		
		
	1	*N(%)*	17 (39.5)	17 (40.5)			
		
	2	*N(%)*	1 (2.3)	2 (4.8)			
		
cg_24	0	*N(%)*	14 (53.8)	11 (45.8)	*U* = 281 *p* = .491		
		
	1	*N(%)*	12 (46.2)	12 (50.0)			
		
	2	*N(%)*	0 (0.0)	1 (4.2)			
		
			LCPT	MR-4	the Mann Whitney *U* test		
ct_0	0	*N(%)*	28 (68.3)	32 (76.2)	*U* = 788 *p* = .392		
		
	1	*N(%)*	12 (29.3)	10 (23.8)			
		
	2	*N(%)*	1 (2.4)	0 (0.0)			
		
ct_3	0	*N(%)*	12 (26.1)	16 (41.0)	*U* = 711 *p* = .060		
		
	1	*N(%)*	28 (60.9)	22 (56.4)			
		
	2	*N(%)*	6 (13.0)	1 (2.6)			
		
ct_12	0	*N(%)*	12 (27.9)	12 (28.6)	*U* = 864.5 *p* = .702		
		
	1	*N(%)*	24 (55.8)	25 (59.5)			
		
	2	*N(%)*	5 (11.6)	5 (11.9)			
		
	3	*N(%)*	2 (4.7)	0 (0.0)			
		
ct_24	0	*N(%)*	5 (19.2)	6 (25.0)	*U* = 277.5 *p* = .470		
		
	1	*N(%)*	12 (46.2)	12 (50.0)			
		
	2	*N(%)*	8 (30.8)	5 (20.8)			
		
	3	*N(%)*	1 (3.8)	1 (4.2)			
		
			LCPT	MR-4	the Mann Whitney *U* test		
ci_0	0	*N(%)*	31 (75.6)	35 (83.3)	*U* = 791 *p* = .362		
		
	1	*N(%)*	9 (22.0)	7 (16.7)			
		
	2	*N(%)*	1 (2.4)	0 (0.0)			
		
ci_3	0	*N(%)*	21 (45.7)	16 (41.0)	*U* = 882 *p* = .883		
		
	1	*N(%)*	19 (41.3)	22 (56.4)			
		
	2	*N(%)*	6 (13.0)	1 (2.6)			
		
ci_12	0	*N(%)*	11 (25.6)	10 (23.8)	*U* = 902 *p* = .992		
		
	1	*N(%)*	25 (58.1)	26 (61.9)			
		
	2	*N(%)*	5 (11.6)	4 (9.5)			
		
	3	*N(%)*	2 (4.7)	2 (4.8)			
		
ci_24	0	*N(%)*	6 (23.1)	5 (20.8)	*U* = 283 *p* = .536		
		
	1	*N(%)*	12 (46.2)	15 (62.5)			
		
	2	*N(%)*	6 (23.1)	3 (12.5)			
		
	3	*N(%)*	2 (7.7)	1 (4.2)			
		
			LCPT	MR-4	the Mann Whitney *U* test		
cv_0	0	*N(%)*	26 (63.4)	27 (65.9)	*U* = 835.5 *p* = .956		
		
	1	*N(%)*	9 (22.0)	5 (12.2)			
		
	2	*N(%)*	6 (14.6)	9 (22.0)			
		
cv_3	0	*N(%)*	26 (57.8)	21 (53.8)	*U* = 838 *p* = .693		
		
	1	*N(%)*	9 (20.0)	8 (20.50			
		
	2	*N(%)*	10 (22.2)	10 (25.6)			
		
cv_12	0	*N(%)*	15 (34.9)	15 (35.7)	*U* = 901 *p* = .985		
		
	1	*N(%)*	17 (39.5)	16 (38.1)			
		
	2	*N(%)*	11 (25.6)	11 (26.2)			
		
cv_24	0	*N(%)*	4 (15.4)	8 (33.3)	*U* = 298 *p* = .759		
		
	1	*N(%)*	19 (73.1)	10 (41.7)			
		
	2	*N(%)*	3 (11.5)	6 (25.0)			
		
			LCPT	MR-4	the Mann Whitney *U* test		
cg_0	0	*N(%)*	38 (92.7)	41 (97.6)	*U* = 818.5 *p* = .297		
		
	1	*N(%)*	3 (7.3)	1 (2.4)			
		
cg_3	0	*N(%)*	40 (87.0)	31 (79.5)	*U* = 830 *p* = .358		
		
	1	*N(%)*	6 (13.0)	8 (20.5)			
		
cg_12	0	*N(%)*	25 (58.1)	23 (54.8)	*U* = 864 *p* = .693		
		
	1	*N(%)*	17 (39.5)	17 (40.5)			
		
	2	*N(%)*	1 (2.3)	2 (4.8)			
		
cg_24	0	*N(%)*	14 (53.8)	11 (45.8)	*U* = 281 *p* = .491		
		
	1	*N(%)*	12 (46.2)	12 (50.0)			
		
	2	*N(%)*	0 (0.0)	1 (4.2)			
		
			LCPT	MR-4	the Mann Whitney *U* test		
g_0	0	*N(%)*	41 (100.0)	41 (97.6)	*U* = 840.5 *p* = .323		
		
	1	*N(%)*	0 (0.0)	1 (2.4)			
		
g_3	0	*N(%)*	37 (82.2)	32 (82.1)	*U* = 877 *p* = .995		
		
	1	*N(%)*	4 (8.9)	4 (10.3)			
		
	2	*N(%)*	4 (8.9)	3 (7.7)			
		
g_12	0	*N(%)*	38 (88.4)	38 (90.5)	*U* = 884.5 *p* = .761		
		
	1	*N(%)*	4 (9.3)	3 (7.2)			
		
	2	*N(%)*	1 (2.3)	1 (2.4)			
		
g_24	0	*N(%)*	23 (88.5)	21 (87.5)	*U* = 309 *p* = .918		
		
	1	*N(%)*	3 (11.5)	3 (12.5)			
		
			LCPT	MR-4	the Mann Whitney *U* test		
i_0	0	*N(%)*	40 (97.6)	41 (97.6)	*U* = 860.5 *p* = .968
		
	1	*N(%)*	1 (2.4)	1 (2.4)	
		
i_3	0	*N(%)*	19 (41.3)	21 (53.8)	*U* = 764 *p* = .206		
		
	1	*N(%)*	15 (32.6)	11 (28.2)			
		
	2	*N(%)*	10 (21.7)	7 (17.9)			
		
	3	*N(%)*	2 (4.3)	0 (0.0)			
		
i_12	0	*N(%)*	12 (27.9)	16 38.1)	*U* = 871 *p* = .975		
		
	1	*N(%)*	26 (60.5)	18 (42.9)			
		
	2	*N(%)*	4 (9.3)	6 (14.3)			
		
	3	*N(%)*	1 (2.3)	2 (4.8)			
		
i_24	0	*N(%)*	10 (38.5)	14 (53.8)	*U* = 269.5 *p* = .370		
		
	1	*N(%)*	12 (46.2)	5 (20.8)			
		
	2	*N(%)*	3 (11.5)	4 (16.7)			
		
	3	*N(%)*	1 (3.8)	1 (4.2)			
		
			LCPT	MR-4	the Mann Whitney *U* test
t_0	0	*N(%)*	41 (100.0)	39 (92.9)	*U* = 799.5 *p* = .083		
		
	1	*N(%)*	0 (0.0)	3 (7.1)			
		
t_3	0	*N(%)*	36 (78.3)	30 (76.9)	*U* = 899.5 *p* = .928		
		
	1	*N(%)*	4 (8.7)	4 (10.3)			
		
	2	*N(%)*	5 (10.9)	5 (12.8)			
		
	3	*N(%)*	1 (2.2)	0 (0.0)			
		
t_12	0	*N(%)*	38 (88.4)	34 (81.0)	*U* = 836.5 *p* = .350		
		
	1	*N(%)*	2 (4.7)	4 (9.5)			
		
	2	*N(%)*	3 (7.0)	3 (7.1)			
		
	3	*N(%)*	0 (0.0)	1 (2.4)			
		
t_24	0	*N(%)*	23 (88.5)	21 (87.5)	*U* = 309 *p* = .918
		
	1	*N(%)*	1 (3.8)	0 (0.0)	
		
	2	*N(%)*	1 (3.8)	3 (12.5)	
		
	3	*N(%)*	1 (3.8)	0 (0.0)	
		
			LCPT	MR-4	the Mann Whitney *U* test		
v_0	0	*N(%)*	36 (87.8)	38 (90.5)	*U* = 838 *p* = .697		
		
	1	*N(%)*	5 (12.2)	4 (9.5)			
		
v_3	0	*N(%)*	42 (91,3)	35 (89.7)	*U* = 884 *p* = .821		
		
	1	*N(%)*	2 (4.3)	3 (7.7)			
		
	2	*N(%)*	2 (4.3)	0 (0.0)			
		
	3	*N(%)*	0 (0.0)	1 (2.6)			
		
v_12	0	*N(%)*	37 (86.0)	40 (95.2)	*U* = 8225 *p* = .160		
		
	1	*N(%)*	5 (11.6)	1 (2.4)			
		
	2	*N(%)*	1 (2.3)	1 (2.4)			
		
v_24	0	*N(%)*	24 (92.3)	23 (95.8)	*U* = 300.5 *p* = .587		
		
	1	*N(%)*	1 (3.8)	1 (4.2)			
		
	2	*N(%)*	1 (3.8)	0 (0.0)			
		
			LCPT	MR-4	the Mann Whitney *U* test		
ptc_0	0	*N(%)*	41 (100.0)	41 (97.6)	*U* = 861 *p* = 1		
		
	1	*N(%)*	0 (0.0)	1 (2.4)			
		
ptc_3	0	*N(%)*	39 (84.8)	31 (79.5)	*U* = 847 *p* = .506		
		
	1	*N(%)*	5 (10.1)	5 (12.9)			
		
	2	*N(%)*	2 (4.3)	3 (7.7)			
		
ptc_12	0	*N(%)*	33 (76.7)	35 (83.3)	*U* = 835 *p* = .391
		
	1	*N(%)*	5 (11.6)	5 (11.9)	
		
	2	*N(%)*	4 (9.3)	2 (4.8)	
		
	3	N*(%)*	1 (2.3)	0 (0.0)	
		
ptc_24	0	*N(%)*	26 (100.0)	24 (100.0)	*U* = 312 *p* = 1		
		
			LCPT	MR-4	the Mann Whitney *U* test
Cd4_0	0	*N(%)*	41 (100.0)	42 (100.0)	*U* = 861 *p* = 1		
		
Cd4_3	0	*N(%)*	39 (84.8)	28 (71.8)	*U* = 784,5 *p* = .163		
		
	1	*N(%)*	5 (10.9)	3 (7.7)			
		
	2	*N(%)*	2 (4.3)	0 (0.0)			
		
Cd4_12	0	*N(%)*	37 (86.0)	35 (83.5)	*U* = 878,5 *p* = .965		
		
	1	*N(%)*	3 (7.0)	4 (9.5)			
		
	2	*N(%)*	3 (7.0)	2 (4.8)			
		
Cd_24	0	*N(%)*	26 (100.0)	24 (100.0)	*U* = 312 *p* = 1		
		
BChS in LCPT and MR-4 groups							
	LCPT	MR-4			
	*M*	*SD*	*M*	*SD*	*U*	*Z*	*P* ^*1*^
BChS _0 (Banff Chronicity Score)	1,20	1,40	0,98	1,22	797,0	-0,62	0,532
BChS_12m (Banff Chronicity Score)	3,23	1,88	3,19	1,71	896,5	-0,06	0,954
BChS _24m (Banff Chronicity Score)	3,77	1,92	3,54	2,19	279,0	-0,65	0,516
BChS _3m (Banff Chronicity Score)	2,30	1,67	2,15	1,41	860,5	-0,33	0,742

ah, arterial hyalinosis; cg, GBM double contours; ct, tubular atrophy; ci, interstitial fibrosis; cv, vascular fibrous intimal thickening; g, glomerulitis; i, interstitial Inflammation; BChS, Banff Chronicity Score; t, tubulitis; v, intimal arteritis; ^1the Mann-Whitney U test.^

### Tac dose and concentration

The LCPT and MR-4 populations required Tac doses of 3.16 ± 1.74 mg and 4.14 ± 1.67 mg at 12 months and 2.48 ± 0.91 mg and 3.2 ± 1.48 mg at 24 months post-transplantation to obtain therapeutic drug trough concentration. Tac doses in the MR-4 group were significantly higher at these time points (*p* = 0.007, *p* = 0.009, respectively), whereas the doses between day 14 and 6 months were comparable (Table 4).

**Table 4:** Tacrolimus dose, whole blood concentration (C_0_) and C/D ratio within 24 months follow-up post-transplant.

Tac Concentration									
	LCPT		MR-4				95% *CI*		
	*M*	*SD*	*M*	*SD*	*t*	*p* ^*1*^	*LL*	*UL*	Cohen’s *d*
14d	12.76	3.95	11.65	3.33	1.62	.109	-0.25	2.46	0.30
1m	11.26	4.30	12.13	4.49	-1.05	.297	-2.52	0.78	0.20
3m	9.62	3.37	8.86	2.91	1.24	.217	-0.45	1.98	0.24
6m	7.81	2.79	7.54	2.47	0.51	.611	-0.78	1.31	0.10
12m	7.09	2.05	6.51	1.78	1.44	.153	-0.22	1.37	0.30
24m	6.38	2.04	6.66	1.88	-0.65	.515	-1.15	0.58	0.14
^1Student’s *t*-test^									
Tac Dose									
	LCPT		MR-4				95% *CI*		
	*M*	*SD*	*M*	*SD*	*t*	*p* ^1^	*LL*	*UL*	Cohen’s *d*
14d	9.49	4.02	10.16	3.36	-0.97	.336	-2.04	0.70	0.18
1m	7.72	3.44	8.99	3.74	-1.88	.063	-2.60	0.07	0.35
3m	5.65	3.19	6.08	2.66	-0.76	.449	-1.57	0.70	0.15
6m	4.37	2.56	4.78	2.12	-0.88	.383	-1.35	0.52	0.18
12m	3.16	1.74	4.14	1.67	-2.77	**.007**	-1.69	-0.28	0.58
24m	2.48	0.90	3.19	1.46	-2.67	**.009**	-1.25	-0.18	0.59
^1 Student’s *t*-test^									
C/D ratio									
	LCPT		MR-4					
	*M*	*SD*	*M*	*SD*	*U*		*Z*		*p* ^*2*^
14d	1.60	1.03	1.25	0.50	1299.0		-1.98		**.048**
1m	1.73	1.13	1.67	1.39	1501.0		-0.54		.589
3m	2.29	1.49	1.87	1.95	1061.5		-1.89		**.059**
6m	2.39	1.56	2.31	3.94	985.5		-1.67		**.095**
12m	2.78	1.35	1.86	1.27	528.5		-4.13		**.000**
24m	2.70	0.75	2.27	0.61	562.0		-2.44		**.015**

2 the Mann-Whitney U test.

The mean Tac concentration remained comparable at every time point during the 24-month observation. The overall mean Tac levels were 7.09 ± 2.05 ng/mL and 6.51 ± 1.78 ng/mL at 12 months, 6.38 ± 2.02 ng/mL and 6.66 ± 1.91 ng/mL at 24 months post-transplantation (*p* = 0.153, *p* = 0.515, respectively) (Table 4).

C_0_/D ratios were significantly higher at 12 and 24 months in the LCPT group than in the MR-4 group and accounted for 2.78 vs. 1.86 *p* = 0.000 and 2.7 vs. 2.27 *p* = 0.015. However, higher C/D ratios persisted throughout the observation period (Table 4).

### Adverse events

The total number of reported AEs was 121 and 141 in the LCPT and MR-4 groups, respectively. The predominant AEs were urinary tract infections, diarrhea, and leukopenia. Detailed treatment-related AEs are summarized in Table 5.

**Table 5:** Adverse events in LCPT and MR-4 group 12 and 24 months posttransplant.

		12m			24m		
		LCPT	MR-4	*p* ^*1*^	LCPT	MR-4	*p* ^*1*^
PVN	N (%)	6 (10.2)	5 (8.9)	p = .837*	2 (4.7)	2 (4.8)	p = 1
UTI	N (%)	19 (32.2)	21 (37.5)	p = .491*	16 (37.2)	18 (42.9)	p = .282*
Pulmonary Aspergillosis	N (%)	0 (0.0)	2 (3.6)	p = .235			
Bronchitis	N (%)	3 (5.1)	1 (1.8)	p = .619	4 (9.3)	3 (7.3)	p = 1
Pneumoniae	N (%)	3 (5.1)	4 (7.1)	p = .712	3 (7.0)	4 (9.5)	p = .713
Sepsis	N (%)	2 (3.4)	9 (16.1)	p = .021*	2 (4.7)	3 (7.1)	p = .676
CMV	N (%)	3 (5.1)	7 (12.5)	p = .196	2 (4.7)	2 (4.8)	p = 1
Diarrhea	N (%)	7 (11.9)	7 (12.5)	p = 1*	7 (16.3)	5 (11.90	p = .757*
CV incident	N (%)	1 (1.7)	1 (1.80	p = 1	2 (4.7)	0 (0.0)	p = .494
Aminotransferase elevation	N (%)	3 (5.1)	5 (8.9)	p = .483	3 (7.1)	2 (4.8)	p = 1
Thrombocytopenia	N (%)	3 (5.1)	2 (3.6)	p = 1	4 (9.3)	3 (7.1)	p = 1
Leukopenia	N (%)	9 (15.3)	13 (23.2)	p = .278*	6 (14.0)	3 (7.1)	p = .483
Neurotoxicity	N (%)	4 (6.8)	9 (16.1)	p = .146*	7 (16.3)	10 (23.8)	p = .427*

PVN, Polyoma virus nephropathy; UTI, urinary tract infection; CMV, cytomegalovirus; CV indicent, cardio-vascualr incident.

1The chi-square or Fisher’s exact test (*The chi^2 test).

Except for a significant difference in sepsis occurrence at 12 months, 3.4% vs. 16.1% (LCPT vs. MR-4; *p* = 0.021, respectively), similar safety profiles were observed. The disparities stemmed primarily from the incidence of sepsis within the first 3 months post-transplantation. Sepsis occurrence at 12 months was correlated with diabetes and graft loss at 12 months and on dialysis in the MR-4 group (*p* = 0.010; *V*_*c*_ = 0.36; *p = 0*.002; *V*_*c*_ = 0.63; respectively). Five patients required once-daily Tac discontinuation (three patients in the LCPT group and two in the MR-4 group). In the LCPT group, two of the three aforementioned patients had to have Tac concentrations optimized and were switched to IR-Tac. In one case, LCPT was discontinued owing to the occurrence of Tac-related AEs. In the MR-4 group, Tac withdrawal in both patients was caused by drug intolerance development. Tac withdrawal rates were similar between groups, 5.1% vs. 3.6%, *p* = 1.

The emergence of NODAT at 12 months was similar, although relatively high at 51.7% vs. 48.2% (LCPT vs. MR-4, chi^2(1), *p* = 0.852, respectively), with onset reported mainly in the first 6 months post-transplantation. The prevalence of cytomegalovirus (CMV) infection showed no considerable differences, although it occurred more often at 12 months in the MR-4 group (5.1% vs. 12.5%; LCPT vs. MR-4, respectively, *p* = 0.196) and was noted mainly within the first 6 months post-transplantation.

## Discussion

This retrospective, real-cohort study aimed to compare the effectiveness and safety profiles of two once-daily prolonged-release Tac formulations in deceased donor KTRs.

The main causes of death and graft loss were cardiovascular complications, infections, and antibody-mediated chronic rejection. Establishing an optimal dosage schedule of Tac to obtain an optimal risk-benefit ratio is of particular importance.

According to European Renal Association–European Dialysis and Transplant Association, patient 5-year survival following the first kidney transplant from a deceased donor accounts for 92%, and kidney allografts account for over 80% [5]. In our study, the 2-year survival of patients and grafts was approximately 90%. Although there was no statistically significant difference in the graft survival between the groups it was superior in the LCPT group. This could have resulted in a greater number of first kidney transplants and shorter dialysis time compared with the MR-4 group. Moreover, a correlation was found between graft outcome and sepsis, with sepsis observed more often in the MR-4 group. None of the patients who experienced steroid-resistant acute rejection (sr-AR) lost the grafts.

Despite the significant advancements, long-term graft survival remains an important issue.

The primary outcome of this study was the assessment of allograft function. We found that renal function assessed using Scr and eGFR estimated at predetermined time points within 24 months of follow-up was comparable between both groups. Our findings are broadly consistent with previously conducted studies, although sparse; similarly, no differences in Scr and eGFR within the LCPT and MR-4 groups were reported at the 3-, 6- and 12-month follow-up [6–8].

In most studies, the frequency of DGF and PNF episodes was independent of the Tac formulation administered [6,7]. We arrive at the same conclusion in our analysis.

The Tac whole blood concentration in this study at various time points did not differ significantly between the groups. Recent studies have suggested that maintenance of Tac concentration at higher levels may be more beneficial than Tac underdosing [9].

In daily practice, C_0_ remains the only indicator of Tac metabolism. Although the association between the trough level of Tac and nephrotoxicity was determined, some authors suggest its appearance despite low C_0_ levels [10]. This may suggest that trough blood levels of Tac did not entirely depict the patient’s exposure to the drug. Some authors suggest the use of the C/D ratio as a more adequate indicator of Tac metabolism to better titrate its dose, as Tac metabolism is said to influence short- and long-term graft survival [11]. Others point to C/D ratio employment to discriminate against patients at high risk of CNI-induced nephrotoxicity (CITN). No treatment is available for CITN; therefore, it is of great importance to minimize this effect.

Our analysis revealed a significantly higher C/D ratio at 12 and 24 months after kidney transplantation in the LCPT population. Higher C/D ratios were also observed at the remaining distribution points. In the MR-4 population, higher nephrotoxicity was reported at 3 months, and the C/D ratio reached lower values. This is consistent with the findings of Thölking et al., where a negative correlation between CITN and a low C/D ratio was noted [12]. Nevertheless, in our study, the patients in the MR-4 group did not have a greater prevalence of CITN at any other time point, despite the lower C/D ratio in comparison with the LCPT population.

The emergence of TCMR and ABMR proved to be comparable in both study arms, although a higher incidence of sr AR was observed in the MR-4 group than in the LCPT group. As the number of biopsies in this study at 24 months was scarce, further studies are needed. Most trials addressed the incidence of TCMR and ABMR as a whole (BPAR) with regard to the implemented Tac formulation. Our research differentiates between TCMR and ABMR and deals with them separately.

In this analysis, no differences between the two cohorts were noted with respect to dnDSA development at any time point. The incidence of dnDSA was comparable to that observed in previous studies, where the cumulative incidence of dnDSAs reached up to 20%, predominantly within the first year post transplantation [13]. Despite the mean Tac C_0_ remaining within the target range, the patient may be exposed to low levels of immunosuppressive drugs, prompting an alloimmune response and dnDSA development. The development of *de novo* donor-specific HLA antibodies is considered a risk factor for graft loss and the incidence of ABMR [13–15].

We observed a higher ah at 3 months in the MR-4 group. This increased prevalence may stem from the greater extent of arterial hyalinosis at baseline. Higher ah was not observed at any other follow-up point. Aggressive treatment with sr-AR in the MR-4 group may have contributed to this finding. CITN in biopsy specimens occurs as interstitial fibrosis (ci), tubular atrophy (ct) and glomerulosclerosis, although nonspecific, and may reflect many other pathologies such as obstructive nephropathy, diabetes, or hypertension. The most characteristic finding indicative of CITN is *de novo* or progression of arterial hyalinosis (ah) [16]. In none of the studies known to the author was increased nephrotoxicity of any of the prolonged-release Tac formulations noted. Some studies have found a trend toward milder interstitial fibrosis and tubular atrophy with MR-4: prolonged-release Tac at 12 months, with no simultaneous differences in arterial hyaline changes.

In our study, more prominent ah and ct parameters were observed in the MR-4 group. Furthermore, the analysis was performed on a small number of samples, and such a tendency was not observed at the remaining time points and must be regarded with caution. Further studies are required to determine the implications of these findings.

In our analysis, the incidence of sepsis was noticeably higher after 12 months of observation in the MR-4 cohort. This may be partly explained by the greater percentage of highly sensitized patients receiving induction therapy in this group. Furthermore, two patients in the MR-4 cohort were diagnosed with sr-AR and required ATG administration. Krąmer et al. did not notice any difference in the C_0_, viral infection, or severe infection prevalence between the prolonged-release Tac and MR-4 groups [17].

In our study, neurotoxicity did not differ significantly in any of the examined groups, although a trend toward lower neurotoxicity in the LCPT group persisted throughout the 24 months of follow-up. Disparities in the pharmacokinetic properties of different Tac formulations administered once daily may arise from a more distal distribution in the gut and lower impact of cytochrome CYP3A5on the metabolism of LCPT compared with MR-4. Some studies have emphasized the probable reduction of Tac-induced neurotoxicity, particularly tremor, during MeltDose technology application. Fructuoso et al. investigated 365 patients and observed a 9% decline in tremor prevalence after conversion from other Tac formulations to LCPT ^8^. The STRATO trial showed less neurotoxicity of LCPT compared with IR-Tac [18].

The research showed similar NODAT incidents in both study arms at 12 months. Notably, the prevalence of pre-transplant diabetes was also relatively high. NODAT occurs in 2% to 53% of solid organ transplant recipients and is attributed to immunosuppressive regimens in up to 74% [19]. Knight and Morris, in their meta-analysis showed that steroid avoidance or withdrawal protocols have the potential to lower NODAT incidence but increase the risk of AR [20]. In our study, solely deceased donor kidney recipients were taken under consideration, and a chronic low dose steroid therapy was maintained (equivalent to 5 mg of prednisone), although in 7% of patients, higher steroid doses were required. The high incidence of NODAT in our study may be partly attributed to the maintenance of Tac levels at the upper limit of the therapeutic range to prevent AR episodes. Moreover, patients with baseline glycosylated hemoglobin of >6% or fasting glucose of >126 mg/dL before transplantation were not excluded from the analysis.

Existing data regarding NODAT are challenging to compare due to inconsistent diagnostic criteria implemented in different trials, from a single registered random glucose of 11.1 mmol/L or greater need for insulin usage over 30 days, leading to overestimation or underestimation of incidence.

CMV infection occurred similarly in both study groups, notwithstanding the trend of higher prevalence in the MR-4 group and the early post-transplant period. Certainly, the unfavorable CMV donor/recipient serostatus in the MR-4 group in three cases combined with a greater induction therapy percentage and an aggressive sr-AR treatment in this cohort contributed to this finding. The available data remain inconsistent with regard to CMV infection in patients with an IR or prolonged-release Tac formulation [17,21].

## Conclusion

In conclusion, our study showed that once-daily prolonged-release Tac formulations are safe and efficient, providing comparable graft outcomes. Nevertheless, further studies are needed to optimize and personalize Tac treatment following kidney transplantation.
